# Personality and Psychological Distress: Statistical Mediation Through Constructive and Destructive Coping in Oncological Patients

**DOI:** 10.3390/jcm15208161

**Published:** 2026-10-20

**Authors:** Robert Jan Łuczyk, Marta Łuczyk, Dorota Weber, Kamil Sikora, Anna Charuta, Anna Pacian

**Affiliations:** 1 Institute of Health Sciences, Faculty of Medical and Health Sciences, University of Siedlce, 08-110 Siedlce, Poland; anna.charuta@uws.edu.pl; 2 Long-Term Care Nursing Department, Chair of Preventive Nursing, Faculty of Health Sciences, Medical University of Lublin, Ul. Chodźki 7, 20-093 Lublin, Poland; marta.luczyk@umlub.edu.pl; 3 Faculty of Health Sciences, Lublin University of Technology, WSEI in Lublin, Projektowa 4, 20-209 Lublin, Poland; dorota.weber@wsei.pl; 4 Department of Internal Medicine and Internal Nursing, Chair of Preventive Nursing, Faculty of Health Sciences, Medical University of Lublin, Ul. Chodźki 7, 20-093 Lublin, Poland; kamil.sikora@umlub.edu.pl; 5 Department of Health Education, Faculty of Health Sciences, Medical University of Lublin, 20-400 Lublin, Poland; anna.pacian@umlub.edu.pl

**Keywords:** personality traits, NEO-FFI, coping styles, Mini-MAC, anxiety, depression, cancer, structural equation modeling, mediation, psycho-oncology

## Abstract

**Background**: Personality traits are established correlates of psychological adjustment to cancer, but their relationships with coping styles, anxiety, and depression require further examination. Constructive coping (fighting spirit, positive reappraisal) and destructive coping (helplessness–hopelessness, anxious preoccupation) are hypothesised intermediates in these relationships. This cross-sectional study examined direct and indirect statistical associations in a structural equation model linking the five NEO-FFI personality traits, both coping styles, and anxiety and depression symptoms in patients with cancer. **Methods**: This multicentre cross-sectional study was conducted between January 2024 to May 2025 in the Department of Clinical Oncology and Radiotherapy and the Department of Oncological Surgery of the Siedlce Oncology Centre, University Hospital No. 4 in Lublin, and University Hospital No. 1 in Lublin, Poland. Statistical analysis was performed on 185 participants. Personality was assessed with the NEO Five-Factor Inventory (NEO-FFI), coping style with the Mini-Mental Adjustment to Cancer (Mini-MAC) scale, and anxiety and depression with the Hospital Anxiety and Depression Scale (HADS). A saturated structural equation model (four multiple regression equations) was estimated, with standardised path coefficients, non-parametric bootstrap (5000 resamples), and 95% bootstrap confidence intervals for direct and indirect effects. All analyses were conducted in R (version 4.2.2) via RStudio (version 2022.12.0), α = 0.05. Personality traits, coping styles, anxiety, and depression were measured concurrently; the specified mediation pathways therefore represent statistical associations rather than evidence of temporal or causal mechanisms. **Results**: The model explained 52% of the variance in depression, 52% in anxiety, 10% in constructive coping, and 9% in destructive coping. Agreeableness was the strongest positive predictor of constructive coping (β = 0.334, *p* = 0.001) and a negative predictor of destructive coping (β = −0.199, *p* = 0.019). Neuroticism and conscientiousness positively predicted destructive coping (β = 0.213, *p* = 0.011; β = 0.218, *p* = 0.028). Constructive coping was negatively associated with both depression (β = −0.247, *p* < 0.001) and anxiety (β = −0.124, *p* = 0.037), while destructive coping was positively associated with both (β = 0.218, *p* = 0.002; β = 0.383, *p* < 0.001). Nominal 95% bootstrap confidence intervals excluded zero for specific indirect associations involving agreeableness and both distress outcomes through both coping styles, and for neuroticism and conscientiousness through destructive coping. **Conclusions**: Constructive and destructive coping were involved in model-specified indirect associations between personality and psychological distress in cancer patients. Agreeableness was associated with lower distress through both coping styles, while neuroticism and conscientiousness showed positive indirect associations through destructive coping. Concurrent measurement, possible construct overlap, unadjusted demographic and clinical factors, and multiple comparisons limit interpretation. These exploratory findings may inform hypotheses for longitudinal studies; they do not establish causal mediation or the effectiveness of coping-focused interventions.

## 1. Introduction

A cancer diagnosis confronts patients with an acute and often prolonged psychological challenge, and a substantial proportion of oncological patients experience clinically significant psychological distress during and after treatment [1,2]. Understanding which patients are most vulnerable to this psychological burden, and through which mechanisms, is central to targeting psycho-oncological support where it is most needed. Anxiety and depression, the two outcomes examined in the present study, are most commonly screened for in oncology settings using the Hospital Anxiety and Depression Scale (HADS), an instrument specifically designed and validated for use with medically ill populations [3,4].

Personality, conceptualised within the Five-Factor Model, is among the most consistently replicated dispositional correlates of anxiety and depression across clinical and non-clinical populations. Meta-analytic evidence shows that neuroticism is robustly associated with elevated risk across virtually all anxiety and depressive disorders, while conscientiousness and, to a lesser and less consistent extent, extraversion and agreeableness are associated with lower risk [5,6]. These associations extend to cancer populations specifically: neuroticism and related distress-prone personality patterns have been linked to greater emotional distress and poorer psychosocial adjustment among patients with lung and breast cancer [7,8,9,10].

Contemporary stress-and-coping models propose that personality is related to the coping strategies a person habitually deploys when facing a threat such as cancer, and that coping may help account for associations between personality and emotional outcomes [11,12,13]. These models motivate hypotheses about indirect relationships, but the temporal and causal processes they propose cannot be established by concurrent measurements. The Mental Adjustment to Cancer framework developed by Watson and colleagues operationalises this coping response along a spectrum from constructive strategies (fighting spirit, positive reappraisal) to destructive strategies (helplessness–hopelessness, anxious preoccupation) [11,12]. Constructive coping has been consistently linked to better quality of life and more favourable psychological outcomes, whereas destructive coping is associated with heightened anxiety and depression [14]. A study of patients with non-small cell lung cancer using an identical tripartite coping classification found markedly higher anxiety and depression scores among patients with predominantly destructive coping compared with those using balanced or constructive strategies [14], directly paralleling the constructive/destructive distinction used in the present study.

Despite this, relatively few studies have examined personality, coping style, and emotional distress within a single model of direct and indirect associations. Most available evidence either examines personality and distress directly [5,6,7] or coping and distress directly [14], leaving the potential intermediary role of coping style comparatively underexplored in oncology populations. Such models can identify patterns warranting longitudinal investigation. Whether coping is a causal mediator and whether coping-focused interventions reduce distress in patients with particular personality profiles require evidence from designs that establish temporal ordering and evaluate intervention effects.

The present study therefore examined a structural equation model in which the five NEO-FFI personality traits (neuroticism, extraversion, openness, agreeableness, conscientiousness) were specified as predictors of constructive and destructive coping (Mini-MAC), which in turn were specified as predictors of anxiety and depression symptoms (HADS) in a sample of patients with cancer. We hypothesised that (1) neuroticism would be positively associated with destructive coping and agreeableness and conscientiousness would be positively associated with constructive coping; (2) destructive coping would be positively associated, and constructive coping negatively associated, with both anxiety and depression; and (3) coping styles would be involved in non-zero statistical indirect associations between personality traits and anxiety and depression. The specified direction follows the theoretical model rather than an observed temporal sequence; mediation terminology refers to the statistical decomposition of concurrent associations [15].

## 2. Materials and Methods

### 2.1. Study Design and Participants

This multicentre cross-sectional study was conducted from January 2024 to May 2025 at three clinical sites in Poland: the Department of Clinical Oncology and Radiotherapy and the Department of Oncological Surgery of the Siedlce Oncology Centre (Siedlce), University Hospital No. 4 in Lublin, and University Hospital No. 1 in Lublin. Statistical analysis was performed on 185 participants.

### 2.2. Measures

#### 2.2.1. NEO Five-Factor Inventory (NEO-FFI)

Personality traits were assessed with the NEO Five-Factor Inventory (NEO-FFI), a 60-item self-report measure of neuroticism, extraversion, openness to experience, agreeableness, and conscientiousness, derived from the original NEO Personality Inventory [16,17]. The Polish adaptation by Zawadzki and colleagues was used [18]. Published psychometric evidence supports the five-factor structure and convergence with other personality measures and observer ratings [18,19]. Internal consistency coefficients reported for the original NEO-FFI range from Cronbach’s α = 0.68 to 0.86 [17,19]. The Polish adaptation has generally satisfactory internal consistency, with lower reliability for openness and agreeableness than for the other dimensions [18]. These findings describe external psychometric evidence, not reliability estimates for the present sample.

#### 2.2.2. Mini-Mental Adjustment to Cancer Scale (Mini-MAC)

Coping style was assessed with the Mini-Mental Adjustment to Cancer (Mini-MAC) scale, a 29-item measure derived from Watson and colleagues’ original Mental Adjustment to Cancer (MAC) scale [11,20] and later refined into its short form [12], validated and normalised in numerous cancer populations, including a Polish sample [21,22,23,24]. The Polish adaptation by Juczyński [25] yields four coping strategies—fighting spirit, positive reappraisal, helplessness–hopelessness, and anxious preoccupation—which are conventionally aggregated into two higher-order styles: a constructive style (fighting spirit and positive reappraisal) and a destructive style (helplessness–hopelessness and anxious preoccupation) [12,24]. The present study used the aggregated constructive and destructive style scores. Published evidence for this adaptation supports convergent validity through expected associations with coping, emotional control, optimism, and cancer-related quality-of-life measures [25]. Reported Cronbach’s α coefficients for the four individual strategies range from 0.87 to 0.92 [25]. These external coefficients apply to the individual strategy scales; they are not estimates of the reliability of the aggregated constructive and destructive scores in the present sample.

#### 2.2.3. Hospital Anxiety and Depression Scale (HADS)

Symptoms of anxiety and depression were assessed with the Hospital Anxiety and Depression Scale (HADS), a 14-item instrument comprising two 7-item subscales (HADS-A for anxiety, HADS-D for depression), each yielding a score from 0 to 21, with higher scores indicating greater symptom severity [3,4]. The standard 14-item Polish HADS was used. The HADS was designed for medically ill populations, including oncology settings [3,4]. In an independent validation study involving 252 Polish gynecologic patients, Cronbach’s α was 0.78 for HADS-A and 0.84 for HADS-D; depressive scores showed convergence with other depression and well-being measures, although several anxiety items had ambiguous factor loadings [26]. These estimates come from a separate validation sample and do not establish reliability in the present study. A researcher-designed questionnaire (metryczka) was used to collect sociodemographic and clinical data, including sex, age, place of residence, education, employment status, illness duration, tumour type, and treatment modality.

Sample-specific internal consistency coefficients (Cronbach’s α or McDonald’s ω) were not available for reporting for the NEO-FFI dimensions, the Mini-MAC composite styles, or the HADS subscales. Accordingly, the published psychometric information above is explicitly distinguished from reliability in this cohort.

### 2.3. Statistical Analysis

Categorical variables were described using frequencies (n) and percentages (%); continuous variables were described using means (M), standard deviations (SD), medians (Me), skewness, and kurtosis. Associations among personality dimensions, coping styles, and anxiety and depression symptoms were assessed using Pearson correlation coefficients. All statistical inference was two-tailed, with the nominal significance level set a priori at α = 0.05. The reported *p*-values and confidence intervals are unadjusted for multiple comparisons.

To test the hypothesised pattern of direct and indirect relationships, structural equation modeling (SEM) was used. The model specification comprised four multiple regression equations. In the first two equations, the five NEO-FFI personality dimensions—neuroticism, extraversion, openness to experience, agreeableness, and conscientiousness—served as predictors of constructive and destructive coping, respectively. In the subsequent two equations, depression and anxiety symptom severity were each modelled simultaneously as a function of the five personality dimensions and both coping styles. For endogenous variables, R^2^ coefficients are reported, indicating the proportion of variance explained by the predictors included in the model. The terms predictor, mediator, direct effect, and indirect effect describe roles or coefficients within the specified statistical model. They do not imply temporal precedence or causal effects. Sex, age, cancer type, illness duration, and treatment modality were not included as covariates in the reported equations.

The model was saturated (df = 0); consequently, global fit indices provided no information about model–data correspondence and were not interpreted. Evaluation instead relied on the individual path parameters. Standardised path coefficients (β), standard errors (SE), z-statistics, *p*-values, and 95% bootstrap confidence intervals are reported for all paths. Specific indirect coefficients were computed as products of the coefficients composing each specified pathway. Inference for these coefficients was based on whether the nominal 95% bootstrap confidence interval excluded zero [27,28]. Such exclusion indicates an indirect statistical association under the specified model, rather than confirmation of a causal mediation mechanism. The tabulated *p*-values accompanying the z-statistics are normal-approximation, z-based *p*-values; they are distinct from inference based on bootstrap confidence intervals. Because products of coefficients can have asymmetric sampling distributions, an indirect coefficient can have a bootstrap confidence interval excluding zero while its z-based *p*-value exceeds 0.05 [27,28]. These are different inferential procedures, so agreement is not guaranteed. The interval criterion is applied consistently to all specific indirect coefficients, while the z-based *p*-values are retained in Appendix A.

Before interpreting the model, the underlying regression assumptions were checked. Multicollinearity among predictors was assessed using the variance inflation factor (VIF); all values remained below 5, with a maximum of 2.05 [29]. Homogeneity of residual variance was assessed visually via residuals-versus-fitted plots and formally via the Breusch–Pagan test; no significant heteroscedasticity was detected (*p* > 0.05) [30]. Potentially influential observations were screened using Cook’s distance; the highest observed value was 0.145, not indicative of unduly influential cases [31]. Normality of residuals was assessed visually using quantile–quantile (Q–Q) plots, while the assumption of multivariate normality was formally tested using Mardia’s test [32].

Because Mardia’s test indicated a significant departure from multivariate normality, parameter uncertainty was estimated using a non-parametric bootstrap procedure based on 5000 resamples with replacement [27,28]. This approach was used to derive 95% confidence intervals for both direct and indirect effects, reducing reliance on the assumption of a normal sampling distribution for products of coefficients. Bootstrapping addresses sampling uncertainty; it does not establish temporal ordering, eliminate confounding or construct overlap, or control the false-positive risk across multiple tested pathways.

All analyses were conducted in R (version 4.2.2) via RStudio (version 2022.12.0).

### 2.4. Ethical Considerations

The study was conducted in accordance with the ethical principles applicable to anonymous, voluntary, and non-interventional research involving human participants, including the principles of respect for autonomy, privacy, confidentiality, and informed consent reflected in the Declaration of Helsinki. Prior to enrolment, all participants received comprehensive verbal and written information regarding the study’s objectives and procedures, the voluntary and anonymous nature of participation, potential risks and benefits, data confidentiality, and their right to withdraw at any time without providing a reason or affecting their medical care. Written informed consent was obtained from each participant before participation in the study.

## 3. Results

### 3.1. Sample Characteristics

The analysed sample comprised 185 patients with cancer (Table 1). Women markedly predominated (77.8%). Almost half of participants (46.0%) were aged 51–61 years, and nearly half (47.6%) had secondary education. The large majority of patients (70.8%) resided in urban areas, and just over half (54.1%) were professionally active. Illness duration was under three months for 18.9% of patients and exceeded one year for 41.6%.

The most frequent tumour diagnoses were cervical cancer (27.0%), endometrial cancer (24.3%), and lung cancer (10.3%); the full distribution across 22 tumour types is presented in Table 2. Regarding treatment received, the large majority of patients underwent chemotherapy (85.4%), just under two in five received radiotherapy (38.9%), and slightly under one in five underwent surgery (18.9%); percentages exceed 100% because treatment modalities were not mutually exclusive (Table 3).

### 3.2. Descriptive Statistics and Correlations Among Study Variables

Descriptive statistics for the five NEO-FFI personality dimensions, HADS anxiety and depression scores, and Mini-MAC constructive and destructive coping scores are presented in Table 4; the full Pearson correlation matrix is presented in Table 5.

Neuroticism correlated positively with both destructive coping (r = 0.226, *p* < 0.01) and, most strongly, with anxiety (r = 0.413, *p* < 0.001) and depression (r = 0.488, *p* < 0.001), and negatively with agreeableness (r = −0.426, *p* < 0.001) and conscientiousness (r = −0.427, *p* < 0.001). Agreeableness showed a pattern of inverse associations with distress: it correlated positively with constructive coping (r = 0.255, *p* < 0.001) and negatively with destructive coping (r = −0.163, *p* < 0.05), anxiety (r = −0.442, *p* < 0.001), and depression (r = −0.483, *p* < 0.001). Destructive and constructive coping were, as expected, strongly and negatively correlated with one another (r = −0.632, *p* < 0.001), and each correlated with anxiety and depression in the expected direction: destructive coping positively (r = 0.544 with anxiety; r = 0.471 with depression, both *p* < 0.001) and constructive coping negatively (r = −0.462 with anxiety; r = −0.480 with depression, both *p* < 0.001). Anxiety and depression were themselves very strongly correlated (r = 0.832, *p* < 0.001), consistent with their shared conceptual basis as indicators of general psychological distress.

### 3.3. Structural Equation Model: Direct Effects

Multicollinearity diagnostics indicated no problematic redundancy among predictors (maximum VIF = 2.05); the Breusch–Pagan test found no significant heteroscedasticity in any of the four regression equations (all *p* > 0.05); and the maximum Cook’s distance (0.145) indicated no unduly influential observations. Mardia’s test indicated a significant departure from multivariate normality, and the 95% confidence intervals reported below were therefore obtained using the 5000-resample non-parametric bootstrap procedure described in Section 2.3. The accompanying z-based *p*-values use a normal approximation and are reported separately from the bootstrap interval criterion.

**Figure 1:**
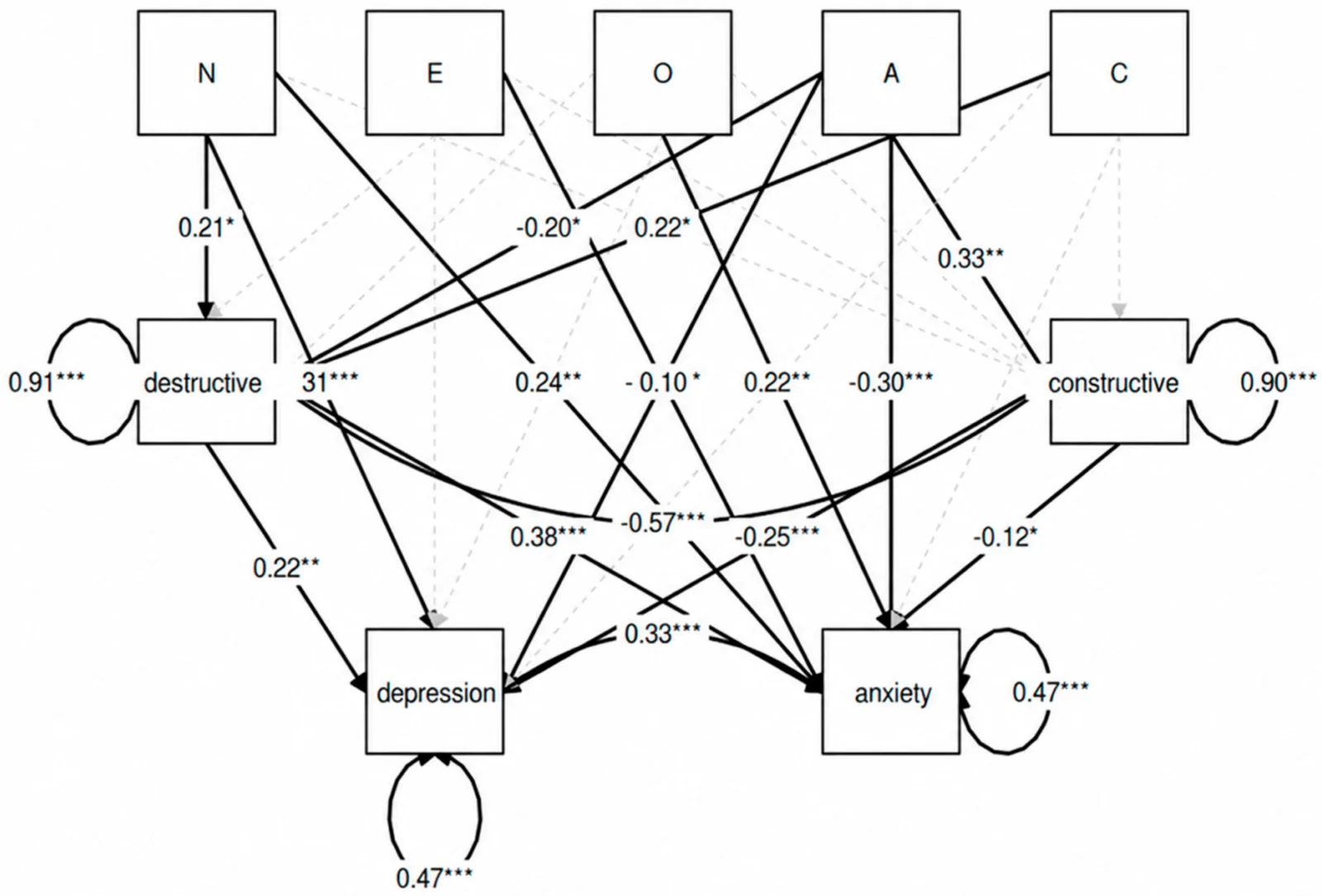
Structural equation model of the relationships between personality traits and depression and anxiety symptoms, with constructive and destructive coping specified as statistical intermediates. Arrows indicate the direction imposed by the regression model, not an established temporal sequence or causal mechanism. The complete direct coefficients, z-based *p*-values, and bootstrap confidence intervals are reported in Table 6. Asterisks denote nominal *p*-value levels: * *p* < 0.05; ** *p* < 0.01; *** *p* < 0.001. N = neuroticism; E = extraversion; O = openness; A = agreeableness; C = conscientiousness.

The full model explained 52% of the variance in depression symptoms and 52% of the variance in anxiety symptoms. For the variables specified as statistical intermediates, the personality dimensions jointly explained 10% of the variance in constructive coping and 9% of the variance in destructive coping. Standardised path coefficients for all 24 direct paths are presented in Table 6 and visualised in Figure 1.

Regarding predictors of coping style, agreeableness was the strongest and most consistent predictor: higher agreeableness predicted greater use of constructive coping (β = 0.334, *p* = 0.001) and less use of destructive coping (β = −0.199, *p* = 0.019). Neuroticism (β = 0.213, *p* = 0.011) and, less intuitively, conscientiousness (β = 0.218, *p* = 0.028) each positively predicted destructive coping, while neither reached significance as a predictor of constructive coping. None of extraversion or openness significantly predicted either coping style.

Regarding predictors of anxiety and depression, both coping styles were significant and substantial predictors of both outcomes in the expected directions: constructive coping was associated with lower depression (β = −0.247, *p* < 0.001) and lower anxiety (β = −0.124, *p* = 0.037), while destructive coping was associated with higher depression (β = 0.218, *p* = 0.002) and, most strongly of all paths in the model, higher anxiety (β = 0.383, *p* < 0.001). After conditioning on both coping styles and the other personality dimensions, neuroticism remained a significant direct predictor of both depression (β = 0.314, *p* < 0.001) and anxiety (β = 0.243, *p* = 0.003), and agreeableness remained a significant direct (negative) predictor of both depression (β = −0.210, *p* = 0.007) and anxiety (β = −0.298, *p* < 0.001). Openness showed a significant direct positive association with anxiety (β = 0.216, *p* = 0.001) despite its non-significant association with either coping style, indicating a conditional association after adjustment for the other model variables, rather than a causal pathway. These coefficients are not adjusted for the demographic or clinical factors listed in Section 2.3. Extraversion and conscientiousness were not significant direct predictors of either depression or anxiety once coping style was accounted for.

### 3.4. Structural Equation Model: Specific Indirect Statistical Associations

Bootstrap-derived specific indirect coefficients are presented in Table 7. Agreeableness showed the most consistent pattern of indirect statistical associations: its coefficient for depression via constructive coping (β = −0.083, 95% CI [−0.165, −0.027]) and via destructive coping (β = −0.043, 95% CI [−0.089, −0.006]) had intervals excluding zero, as did its coefficients for anxiety via constructive coping (β = −0.041, 95% CI [−0.098, −0.003]) and via destructive coping (β = −0.076, 95% CI [−0.146, −0.012]). This pattern links higher agreeableness, more constructive and less destructive coping, and lower distress within the specified cross-sectional model; it does not establish that coping caused the lower distress.

Neuroticism showed an indirect statistical association with both depression (β = 0.046, 95% CI [0.007, 0.113]) and anxiety (β = 0.081, 95% CI [0.018, 0.174]) via destructive coping, but not via constructive coping. Conscientiousness showed a similar indirect statistical association with depression (β = 0.047, 95% CI [0.004, 0.114]) and anxiety (β = 0.083, 95% CI [0.011, 0.181]) via destructive coping—a counter-intuitive finding given conscientiousness’s typically protective role in the broader personality literature, discussed further in Section 4. For these neuroticism and conscientiousness pathways, the nominal bootstrap intervals excluded zero. The intervals for extraversion and openness included zero for both outcomes through both coping styles. All findings use the same interval criterion and remain exploratory because the intervals are unadjusted for multiplicity.

## 4. Discussion

This study examined model-specified indirect statistical associations involving coping style in the relationship between personality traits and psychological distress in patients with cancer. Four findings warrant discussion. First, the model accounted for a substantial proportion of variance in both depression (52%) and anxiety (52%), underscoring that the combination of personality and coping style captures a large share of what distinguishes more from less distressed patients. Second, agreeableness emerged as the personality trait with the most consistent inverse associations with distress, with negative indirect associations with both depression and anxiety through both coping styles. Third, destructive coping was a markedly stronger predictor of anxiety than constructive coping was, and stronger than any personality trait’s direct effect on either outcome, identifying a strong conditional statistical association within this model, rather than a proximal causal driver of distress. Fourth, and most unexpectedly, conscientiousness—usually a protective trait in the general personality literature—was associated with more, not less, destructive coping and, through this pathway, indirectly with more depression and anxiety. All four findings concern concurrent, unadjusted associations and should be interpreted within the methodological limitations discussed below.

The pattern of indirect statistical associations involving agreeableness aligns with its emerging recognition as a protective factor for depression and anxiety across both clinical and non-clinical populations [5,6,33,34]. Agreeable individuals, characterised by interpersonal warmth, cooperativeness, and trust, may be more inclined to accept support from clinicians and family, to engage collaboratively with treatment, and to interpret the cancer experience through a lens of fighting spirit and positive reappraisal rather than helplessness—a possible theoretical account of the nominally significant paths from agreeableness to both coping styles in the present model. This is consistent with meta-analytic evidence that agreeableness is inversely associated with depressive symptoms even after accounting for neuroticism [6]. These possible behavioural explanations were not directly tested, and the present results do not establish a mechanism by which agreeableness affects distress.

The strength of destructive coping as a predictor of anxiety (β = 0.383)—larger than any personality path in the model—echoes findings from lung cancer populations using the identical constructive/destructive Mini-MAC framework, where patients with predominantly destructive coping reported markedly higher anxiety and depression than those with balanced or constructive coping [14], and from studies showing that coping style and personality jointly explain far more variance in cancer-related anxiety than demographic or disease-related factors [9]. In the present study, this association and the corresponding indirect coefficients identify a pattern that warrants further investigation. They do not demonstrate that destructive coping is an active mechanism translating personality into symptoms. Concurrent distress could influence reported coping, and shared anxiety-related content between Mini-MAC anxious preoccupation and HADS-A could contribute to the strength of the association. The findings motivate prospective and intervention studies rather than establish the benefit of modifying coping.

The positive association between conscientiousness and destructive coping, and the corresponding indirect statistical associations with depression and anxiety, runs counter to conscientiousness’s typically protective role in the broader mental-health literature [5,6]. One plausible interpretation, consistent with the anxious-preoccupation component of destructive coping in the Mini-MAC framework, is that highly conscientious patients—oriented toward control, planning, and diligent adherence to treatment—may experience the loss of control inherent to a cancer diagnosis as especially threatening, channelling their characteristic vigilance into anxious preoccupation with the disease rather than fatalistic disengagement. This interpretation is speculative and should be tested directly, for example, by examining whether the conscientiousness–destructive coping association is specific to the anxious-preoccupation subscale rather than the helplessness–hopelessness subscale of the Mini-MAC. Two further considerations counsel particular caution before this finding is interpreted as a general personality–coping association. First, the association, although nominally statistically significant, was modest in size (β = 0.218) and derives from a single cross-sectional sample dominated by gynaecological cancer patients (Section Strengths and Limitations); it could reflect a pattern specific to this clinical and demographic context—for instance, to the particular treatment demands or illness trajectory of cervical and endometrial cancer—rather than a general property of conscientiousness in oncology populations. Second, because conscientiousness was tested as one of five simultaneous personality predictors across four outcome equations, the possibility that this particular path represents a chance finding amid multiple comparisons cannot be excluded on the basis of the present data alone. Independent replication, ideally in a sample with a more balanced tumour-type distribution and correction for multiple comparisons, is needed before this association is interpreted as a robust or generalisable feature of the personality–coping relationship in cancer patients.

Clinically, the observed associations suggest that coping style may be relevant alongside personality when assessing psychological distress in oncology care. Nurses and other clinicians who observe possible maladaptive coping [2,35] may consider anxious preoccupation and helplessness–hopelessness as reasons to assess distress and the need for psychological support. The present cross-sectional model does not show that these coping patterns cause distress or that personality-tailored, coping-focused interventions will improve outcomes; those hypotheses require prospective evaluation.

### Strengths and Limitations

Strengths of this study include a specified model of direct and indirect statistical associations, bootstrap estimation of confidence intervals [27,28], regression diagnostics [29,30,31,32], and established instruments with published psychometric evidence [3,4,11,12,16,17,18,19,24,25,26]. Several limitations should be noted. Personality, coping, anxiety, and depression were measured concurrently. The ordering personality → coping → distress was imposed by the theoretical model and was not observed over time. Reverse or reciprocal associations, such as distress influencing reported coping or personality, cannot be excluded. Cross-sectional indirect coefficients can also differ substantially from the parameters of the corresponding longitudinal process [15]. Accordingly, a bootstrap interval excluding zero indicates a statistical association under the specified model, not evidence that coping temporally or causally mediates an effect of personality. A saturated specification (df = 0) cannot evaluate overall model fit against alternative structures; bootstrapping does not resolve this design limitation.

There is potential conceptual overlap between Mini-MAC anxious preoccupation, which contributes to the destructive coping composite, and HADS-A. Anxious preoccupation assesses a cancer-specific adjustment response characterised by persistent disease-related worry and threat-focused appraisal; HADS-A assesses the severity of anxiety symptoms more generally in medically ill patients [3,12,25]. The constructs differ in their referent and intended role, but both include anxiety-related cognitive and emotional content. Their partial overlap, concurrent self-report measurement, and a possible shared negative-affect component may inflate the destructive coping–anxiety association and its corresponding indirect coefficients. The observed association therefore cannot be assumed to represent a distinct coping mechanism. We did not separately model the anxious-preoccupation and helplessness–hopelessness components or establish discriminant validity between the coping composite and HADS-A. Future studies should examine these components separately and use temporally separated or multimethod measurements.

Women constituted 77.8% of the sample, and cervical and endometrial cancer accounted for 51.3% of diagnoses. This composition limits generalisability and makes sex and cancer type closely related in the sample. Sex and age may be associated with differences in coping and symptom reporting; cancer type, disease duration, and treatment modality may be associated with different illness demands, treatment burdens, and phases of psychological adjustment. These characteristics were measured but were not included as covariates in the reported model. They may confound, attenuate, strengthen, or partly account for the estimated personality–coping and coping–distress associations and their products; the direction and magnitude of any such influence cannot be determined from the reported analyses. In particular, the conscientiousness–destructive coping association may depend on the demographic and clinical composition of this sample. Saturation does not prevent covariate adjustment and is not a justification for omitting these variables. The reported coefficients should therefore not be interpreted as independent of sex, age, cancer type, disease duration, or treatment modality. Covariate-adjusted analyses and appropriately designed replication are needed to evaluate robustness and possible effect modification.

Multiplicity is a further limitation. The reported analyses include 36 distinct pairwise correlations, 24 direct regression paths, and 20 specific indirect pathways. The *p*-values and 95% bootstrap confidence intervals are nominal and unadjusted; these tests are related, and pointwise intervals do not control the probability of false-positive findings across the full set of comparisons. Thus, individual results close to conventional thresholds, including some conscientiousness and other indirect associations, may be false-positive findings. An interval excluding zero should not be described as confirmation of a pathway. All individual-path findings are exploratory and require independent replication. Future confirmatory studies should specify a limited set of primary hypotheses and an appropriate multiplicity strategy.

Finally, the reliability and validity evidence cited in Section 2.2 comes from previous studies or instrument documentation. Sample-specific internal consistency estimates were unavailable for reporting, and the reliability of the Mini-MAC composite styles cannot be inferred from published coefficients for their component strategies. Measurement error and limited separation of the measured constructs may affect the estimated associations. The external psychometric evidence therefore supports instrument selection but does not establish reliability or discriminant validity in this cohort.

## 5. Conclusions

Constructive and destructive coping were involved in model-specified indirect statistical associations between personality traits and psychological distress in this sample of patients with cancer. Agreeableness showed negative indirect associations with both outcomes through both coping styles, whereas neuroticism and conscientiousness showed positive indirect associations through destructive coping. These concurrent associations do not establish causal mediation or the direction of influence. Potential overlap between Mini-MAC anxious preoccupation and HADS-A, demographic and clinical confounding, unavailable sample-specific reliability estimates, and unadjusted multiple comparisons further limit their interpretation. The conscientiousness finding requires particular caution and independent replication. Longitudinal studies with appropriate covariate adjustment and multiplicity control are needed before these patterns can inform claims about mechanisms or intervention effectiveness.

## Figures and Tables

**Table 1:** Sociodemographic and clinical characteristics of the study group (n = 185).

Characteristic	Category	n	%
Sex	Female	144	77.84
	Male	41	22.16
Age	<25 years	10	5.41
	25–40 years	8	4.32
	41–50 years	47	25.41
	51–61 years	85	45.95
	62–72 years	28	15.14
	≥73 years	7	3.78
Place of residence	Urban	131	70.81
	Rural	54	29.19
Education	Primary	14	7.57
	Vocational	37	20.00
	Secondary	88	47.57
	Higher	46	24.86
Employment status	Not working	85	45.95
	Working	100	54.05
Illness duration (months)	<3	35	18.92
	3–6	59	31.89
	6–12	14	7.57
	>12	77	41.62

**Table 2:** Distribution of tumour types in the study group.

Tumour Type	n	%
Pharyngeal or oesophageal cancer	3	1.62
Endometrial cancer	45	24.32
Cervical cancer	50	27.03
Prostate cancer	1	0.54
Ovarian cancer	6	3.24
Oral cavity cancer	7	3.78
Colorectal cancer	5	2.70
Bone cancer	1	0.54
Uterine cancer (other)	4	2.16
Brain cancer	2	1.08
Renal cancer	1	0.54
Anal cancer	16	8.65
Colon cancer	2	1.08
Breast cancer	9	4.86
Bladder cancer	3	1.62
Lung cancer	19	10.27
Skin cancer	2	1.08
Leukaemia	1	0.54
Liver cancer	3	1.62
Gastric cancer	4	2.16
Multiple myeloma	1	0.54

**Table 3:** Type of received therapy.

Treatment Received	n	%
Surgery	35	18.92
Chemotherapy	158	85.41
Radiotherapy	72	38.92

Note: Percentages do not sum to 100% because patients could receive more than one treatment modality.

**Table 4:** Descriptive statistics for personality traits, anxiety, depression, and coping styles.

Variable	M	SD	Median	Skew	Kurt
1. Neuroticism	24.10	6.68	25	−0.15	−0.41
2. Extraversion	26.70	5.68	26	0.02	0.61
3. Openness	24.72	5.76	23	0.43	0.08
4. Agreeableness	28.39	6.45	30	−0.35	−0.59
5. Conscientiousness	31.74	6.78	31	−0.27	−0.14
6. Anxiety	13.18	5.13	15	−0.38	−1.07
7. Depression	11.94	5.63	14	−0.45	−1.06
8. Destructive coping	31.39	8.85	33	−0.33	−1.06
9. Constructive coping	43.45	6.69	43	0.00	−0.97

**Table 5:** Correlation statistics for personality traits, anxiety, depression, and coping styles.

#	Variable	1	2	3	4	5	6	7	8	9
1	Neuroticism	—	−0.241 **	−0.169 *	−0.426 ***	−0.427 ***	0.413 ***	0.488 ***	0.226 **	−0.08
2	Extraversion		—	0.165 *	0.223 **	0.324 ***	−0.07	−0.149 *	−0.14	0.145 *
3	Openness			—	0.196 **	0.09	0.13	0.05	−0.04	−0.06
4	Agreeableness				—	0.665 ***	−0.442 ***	−0.483 ***	−0.163 *	0.255 ***
5	Conscientiousness					—	−0.299 ***	−0.406 ***	−0.04	0.14
6	Anxiety						—	0.832 ***	0.544 ***	−0.462 ***
7	Depression							—	0.471 ***	−0.48 ***
8	Destructive coping								—	−0.632 ***
9	Constructive coping									—

*** *p* < 0.001; ** *p* < 0.01; * *p* < 0.05.

**Table 6:** Standardized direct path coefficients in the specified statistical mediation model (n = 185).

Predictor	Outcome	β	SE	z	*p* (z)	95% Boot CI [LL, UL]
Neuroticism	Constructive coping	0.024	0.082	0.290	0.772	[−0.138, 0.184]
Extraversion	Constructive coping	0.133	0.085	1.561	0.119	[−0.027, 0.306]
Openness	Constructive coping	−0.132	0.078	−1.693	0.090	[−0.293, 0.015]
Agreeableness	Constructive coping	0.334	0.102	3.281	0.001	[0.140, 0.543]
Conscientiousness	Constructive coping	−0.109	0.111	−0.982	0.326	[−0.331, 0.108]
Neuroticism	Destructive coping	0.213	0.084	2.537	0.011	[0.054, 0.385]
Extraversion	Destructive coping	−0.116	0.073	−1.589	0.112	[−0.264, 0.020]
Openness	Destructive coping	0.038	0.072	0.524	0.600	[−0.095, 0.189]
Agreeableness	Destructive coping	−0.199	0.085	−2.344	0.019	[−0.373, −0.032]
Conscientiousness	Destructive coping	0.218	0.099	2.200	0.028	[0.033, 0.422]
Constructive coping	Depression	−0.247	0.060	−4.101	<0.001	[−0.370, −0.133]
Destructive coping	Depression	0.218	0.072	3.031	0.002	[0.077, 0.359]
Constructive coping	Anxiety	−0.124	0.060	−2.082	0.037	[−0.247, −0.013]
Destructive coping	Anxiety	0.383	0.073	5.237	<0.001	[0.248, 0.529]
Neuroticism	Depression	0.314	0.073	4.325	<0.001	[0.178, 0.464]
Extraversion	Depression	0.055	0.060	0.918	0.359	[−0.062, 0.176]
Openness	Depression	0.137	0.068	2.014	0.044	[−0.004, 0.262]
Agreeableness	Depression	−0.210	0.078	−2.701	0.007	[−0.357, −0.059]
Conscientiousness	Depression	−0.120	0.085	−1.411	0.158	[−0.284, 0.050]
Neuroticism	Anxiety	0.243	0.081	3.013	0.003	[0.088, 0.404]
Extraversion	Anxiety	0.100	0.053	1.882	0.060	[0.000, 0.208]
Openness	Anxiety	0.216	0.067	3.214	0.001	[0.077, 0.342]
Agreeableness	Anxiety	−0.298	0.080	−3.733	<0.001	[−0.453, −0.142]
Conscientiousness	Anxiety	−0.017	0.068	−0.251	0.802	[−0.144, 0.118]

Note. β = standardised coefficient; SE = standard error; *p* = nominal z-based normal-approximation *p*-value; 95% Boot CI [LL, UL] = 95% confidence interval (Lower Level, Upper Level) based on 5000 bootstrap resamples. The *p*-values and confidence intervals arise from different inferential procedures and are reported separately; neither is adjusted for multiple comparisons. Coefficients represent conditional statistical associations, not causal effects.

**Table 7:** Bootstrap estimates of model-specified indirect statistical associations between personality traits and depression and anxiety through constructive and destructive coping (n = 185).

Specified Indirect Pathway	β	SE	z	*p* (z)	95% Boot CI [LL, UL]
N → Constructive → Depression	−0.006	0.021	−0.275	0.783	[−0.052, 0.033]
E → Constructive → Depression	−0.033	0.023	−1.412	0.158	[−0.086, 0.006]
O → Constructive → Depression	0.033	0.023	1.443	0.149	[−0.003, 0.086]
A → Constructive → Depression	−0.083	0.036	−2.321	0.020	[−0.165, −0.027]
C → Constructive → Depression	0.027	0.030	0.893	0.372	[−0.027, 0.095]
N → Destructive → Depression	0.046	0.028	1.666	0.096	[0.007, 0.113]
E → Destructive → Depression	−0.025	0.020	−1.243	0.214	[−0.075, 0.004]
O → Destructive → Depression	0.008	0.018	0.464	0.642	[−0.020, 0.052]
A → Destructive → Depression	−0.043	0.021	−2.032	0.042	[−0.089, −0.006]
C → Destructive → Depression	0.047	0.028	1.685	0.092	[0.004, 0.114]
N → Constructive → Anxiety	−0.003	0.011	−0.259	0.796	[−0.029, 0.017]
E → Constructive → Anxiety	−0.017	0.013	−1.259	0.208	[−0.047, 0.004]
O → Constructive → Anxiety	0.016	0.012	1.327	0.184	[−0.003, 0.045]
A → Constructive → Anxiety	−0.041	0.024	−1.724	0.085	[−0.098, −0.003]
C → Constructive → Anxiety	0.014	0.016	0.829	0.407	[−0.015, 0.051]
N → Destructive → Anxiety	0.081	0.040	2.044	0.041	[0.018, 0.174]
E → Destructive → Anxiety	−0.044	0.032	−1.402	0.161	[−0.117, 0.007]
O → Destructive → Anxiety	0.015	0.030	0.491	0.623	[−0.036, 0.084]
A → Destructive → Anxiety	−0.076	0.034	−2.268	0.023	[−0.146, −0.012]
C → Destructive → Anxiety	0.083	0.043	1.930	0.054	[0.011, 0.181]

Note. N = neuroticism; E = extraversion; O = openness; A = agreeableness; C = conscientiousness. β = standardised indirect coefficient (product of path coefficients); *p* = nominal z-based normal-approximation *p*-value; 95% Boot CI [LL, UL] = nominal 95% bootstrap confidence interval based on 5000 resamples. The bootstrap interval criterion described in Section 2.3 is applied to every indirect coefficient. An interval excluding zero supports a non-zero statistical indirect association within the model, including when the accompanying z-based *p*-value exceeds 0.05. The two procedures need not agree because the product-of-coefficients distribution can be asymmetric [27,28]. These pointwise intervals are not adjusted for multiple comparisons and do not confirm causal mediation.

**Figure A1:**
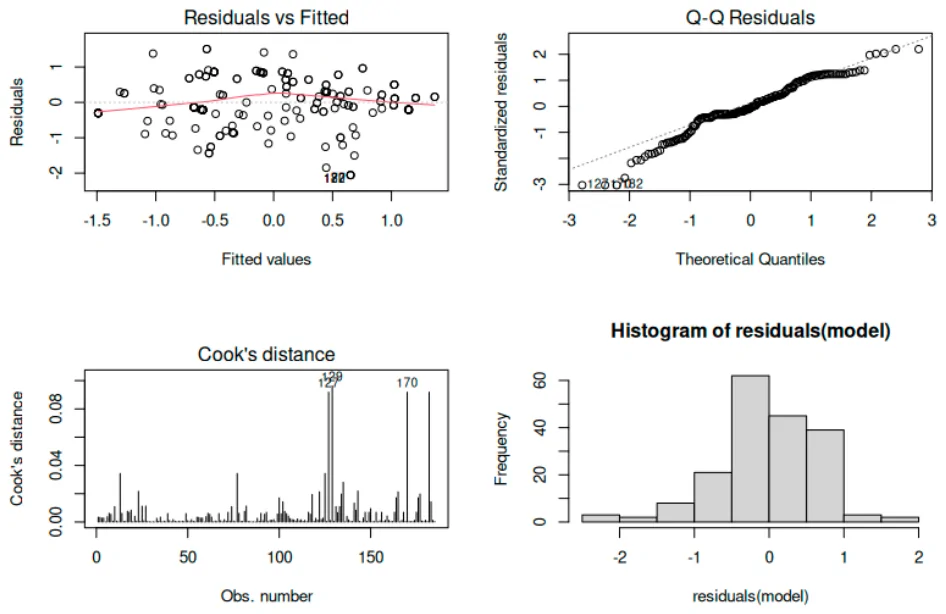
Regression diagnostics for the equation predicting anxiety.

**Figure A2:**
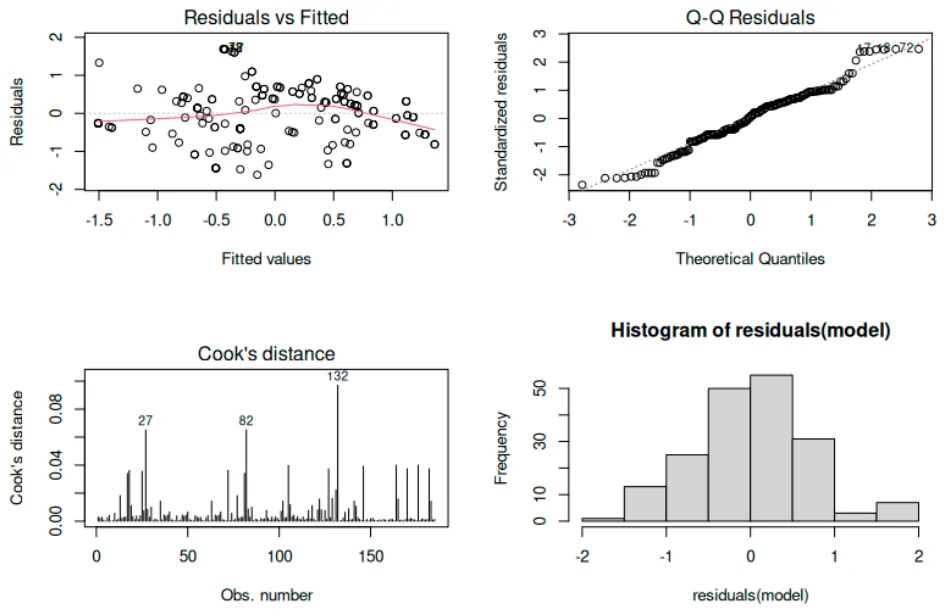
Regression diagnostics for the equation predicting depression.

**Figure A3:**
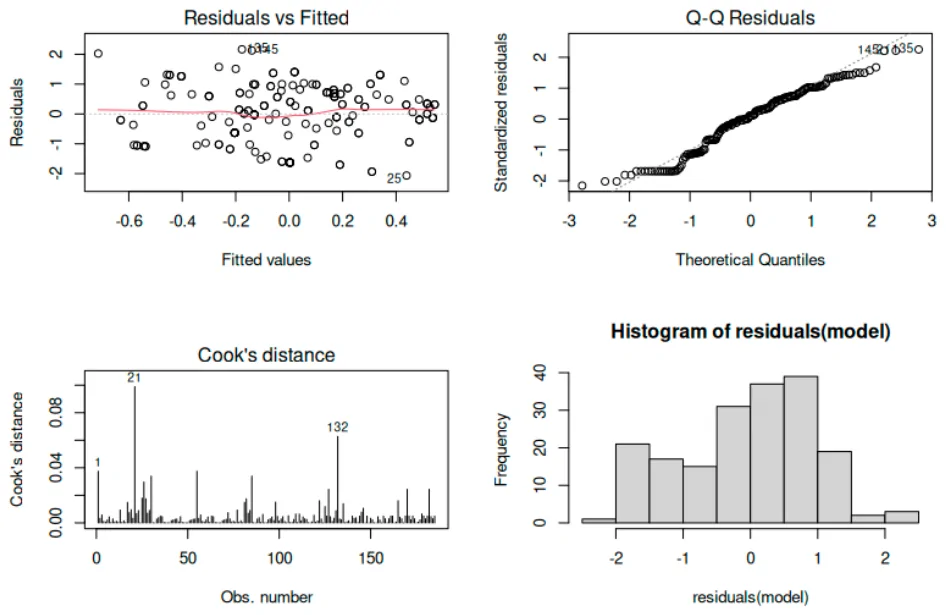
Regression diagnostics for the equation predicting destructive coping.

**Figure A4:**
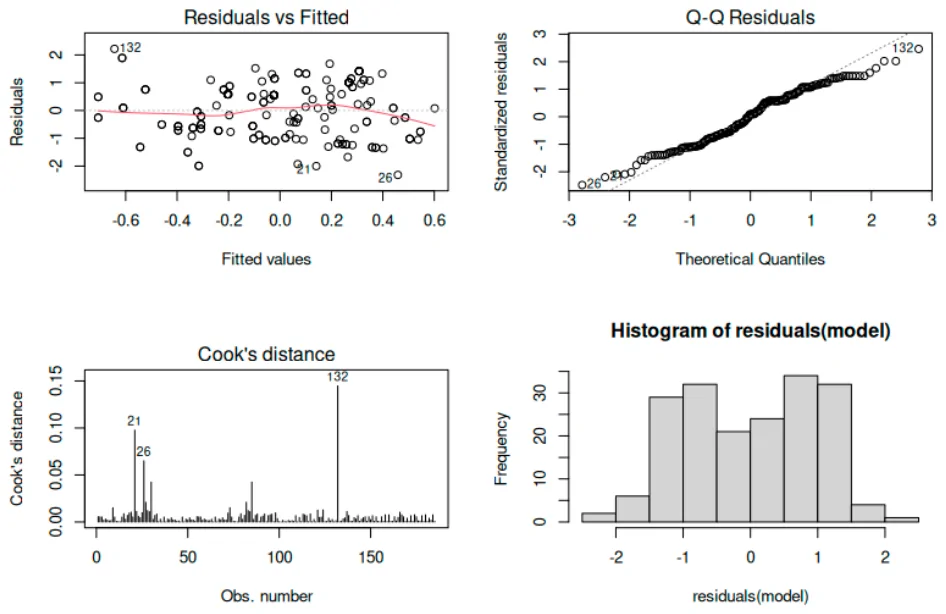
Regression diagnostics for the equation predicting constructive coping.

## Data Availability

The data supporting the findings of this study are available from the corresponding author upon reasonable request, subject to ethical and institutional restrictions protecting participant confidentiality.

## Appendix A. Supplementary Regression Diagnostics

The following figures present residuals-vs-fitted, Q–Q, Cook’s distance, and residual histogram diagnostics for each of the four regression equations comprising the structural equation model, supporting the assumption checks summarised in Section 2.3 and Section 3.3.
